# Racial differences in symptom management approaches among persons with radiographic knee osteoarthritis

**DOI:** 10.1186/1472-6882-12-86

**Published:** 2012-07-06

**Authors:** Shibing Yang, Rachel Jawahar, Timothy E McAlindon, Charles B Eaton, Kate L Lapane

**Affiliations:** 1 Department of Epidemiology and Community Health, Virginia Commonwealth University, Richmond, VA, USA; 2 Department of Rheumatology, Tufts Medical Center, Boston, MA, USA; 3 Departments of Family Medicine and Epidemiology, Warren Alpert Medical School, Brown University, Providence, RI, USA; 4 Center for Primary Care and Prevention, Memorial Hospital of Rhode Island, Pawtucket, RI, USA

**Keywords:** Osteoarthritis, Race, Pain, Complementary and alternative medicine

## Abstract

**Background:**

The extent to which racial differences exist in use of treatments for osteoarthritis (OA) is debatable. The purpose of this study was to describe the differences between African Americans (AA) and Caucasian Americans (CA) in using treatment approaches to manage symptoms among individuals with radiographic-confirmed knee OA.

**Methods:**

A cross-sectional study was conducted. Using data from the Osteoarthritis Initiative, we identified 508 AA and 2,075 CA with radiographic tibiofemoral OA in at least one knee. Trained interviewers asked questions relating to current OA treatments including seven CAM therapy categories—alternative medical systems, mind-body interventions, manipulation and body-based methods, energy therapies, and three types of biologically based therapies, as well as conventional medications. We categorized participants as: conventional medication only users, CAM only users, users of both and users of neither. Multinomial logistic regression models adjusting for sociodemographics and clinical/functional factors provided estimates of the association between race and treatment use.

**Results:**

Overall, 16.5% of AA and 24.2% of CA exclusively used CAM to treat OA, 25.0% of AA and 23.8% of CA used CAM in conjunction with conventional medications, and 24.8% of AA and 14.6% of CA exclusively used conventional medications. After control for sociodemographic and clinical factors, AA were less likely than CA to use CAM therapies alone (adjusted odds ratio (OR) of using CAM alone relative to no CAM or conventional treatments: 0.68, 95% confidence interval (CI): 0.48–0.96) or with conventional medications (adjusted OR relative to no CAM or conventional treatments: 0.59, 95%CI: 0.42–0.83). However, no differences in use of conventional medications alone were observed after adjustment of covariates.

**Conclusion:**

CAM use is common among people with knee OA, but is less likely to be used by AA relative to CA. For effective CAM therapies, targeted outreach to underserved populations including education about benefits of various CAM treatments and providing accessible care may attenuate observed disparities in effective CAM use by race.

## Background

Osteoarthritis (OA) of the knee is the most common type of OA and 12–16% of Americans older than 60 years suffer from this ailment [1,2]. Knee OA has detrimental effects on individuals’ physical function and quality of life and is the leading cause of disability among non-institutionalized adults in the United States [3,4]. Advanced OA accounts for the majority of knee joint replacement surgeries among Medicare recipients [5].

Current guidelines recommend treatment of OA with both pharmacological and non-pharmacological methods, consisting of pain management using acetaminophen or non-steroidal anti-inflammatory drugs (NSAIDs), combined with patient education, exercise, and/or weight loss [6]. Although not part of the guidelines, complementary and alternative medicine (CAM) is increasingly used among persons with OA, possibly due to inadequate healing effects or more adverse effects from conventional medications [7-9], or due to cultural influences which make some race/ethnicity groups favor CAM [10,11]. Indeed, arthritis is the most commonly cited reason for using CAM [12].

The extent to which racial differences exist in use of treatments for OA is debatable. Studies of racial differences in use of CAM therapies among persons with arthritis have been inconsistent [13,14]. In a population-based sample with self-reported arthritis, Caucasian Americans (CA) were 50% more likely than African Americans (AA) to have used CAM [13], while another study with arthritis patients found that AA were more likely to report CAM use than CA (89.1% versus 77.7%) [14]. While understanding differences in CAM use by race is hampered by the lack of systematic CAM definitions, as well as details regarding specific CAM treatments, studies have consistently shown that AA are less likely than CA to use biologically based supplements [13]. Different explanations for racial differences in CAM use have been posited. AA may have developed healing traditions using herbs and other substances owing to lack of access to even rudimentary medical care [15]. It is also possible that differences in disease severity may have accounted for racial differences in the use of CAM therapies [16,17]. Similar to studies on CAM use, studies on racial differences in use of conventional medications for OA also yielded inconsistent results, with some studies [18,19], but not all [17], reporting greater use of conventional medications among CA relative to AA.

The Osteoarthritis Initiative (OAI) is a multi-center, prospective observational study, aimed to examine the natural history of knee OA and to identify risk factors for incidence and progression of knee OA [20]. The availability of the OAI data offers a unique opportunity to evaluate racial differences in OA treatments. First, unlike previous reports [13,14,18,19], the OAI offers a population with radiographic confirmation of OA. Second, the detailed assessments of knee specific pain, quality of life and functioning indicators permit the adjustment for disease severity. Using baseline data from OAI, we sought to describe the differences between CA and AA in using treatment approaches to manage symptoms of knee OA. As our secondary objective, we further identified correlates of therapy choice among CA and AA from sociodemographic and clinical characteristics.

## Methods

The study protocol was approved by the Institutional Review Boards of Virginia Commonwealth University and the Memorial Hospital of Rhode Island. Because publicly-available data were used for this study, the Institutional Review Boards waived the need for documentation of informed consent.

### Study subjects

We used publicly available data from the OAI (#AllClinical00, V0.2.2). From February 2004 to May 2006, four study sites (i.e., Baltimore, MD; Columbus, OH; Pittsburgh, PA; and Pawtucket, RI.) recruited 4,796 participants either with established knee OA or at high risk for developing knee OA [20]. Participants were followed for up to four years.

Participants had a standing posterior-anterior radiographic examination on both knees at enrollment. Using the “fixed-flexion” knee radiography protocol, knees were flexed to 20–30° and feet were internally rotated 10° [21]. An Osteoarthritis Research Society International (OARSI) atlas osteophyte grade greater than or equal to I (equivalent to Kellgren and Lawrence grade ≥ 2) [22] was considered evidence of radiographic knee OA. We included participants with radiographic-confirmed OA in at least one knee. Since only 38 and 58 persons self-identified as Hispanics and “other race”, respectively, we were unable to include them in further race/ethnicity specific analyses. The final analytic sample included 508 participants who self-identified as AA and 2,075 who self-identified as CA.

### Treatment approaches

The National Center for Complementary and Alternative Medicine defined seven broad categories as CAM use including: 1) alternative medical systems (acupuncture, acupressure, homeopathy and others); 2) mind-body interventions (yoga/Tai Chi/Chi Gong/pilates, spiritual activities, relaxation therapy, meditation, deep breathing or visualization); 3) manipulation and body-based methods (chiropractic and massage); 4) energy therapies (copper bracelets or magnets); 5) biologically based topical therapies (rubs, lotions, liniments, creams or oils, capsaicin); 6) biologically based diet; and 7) biologically based supplements (herbals, glucosamine, chondroitin, vitamins/minerals, methylsulfonylmethane, S-adenosylmethionine) [23]. In OAI, a series of questions specifically asked about whether CAM approaches for arthritis or joint pain were used in the past 30 days (for methylsulfonylmethane and S-adenosylmethionine), past 6 months (for glucosamine and chondroitin), or past 12 months (for all other CAM therapies) and how frequently these therapies were used. A participant was considered a CAM user, if he/she self-reported using glucosamine and chondroitin “every day or nearly every day”, taking methylsulfonylmethane and S-adenosylmethionine “more than half days of the past month”, or “currently” using any of other types of CAM therapies.

Participants were asked “During the past 30 days, have you used any of the following medications for joint pain or arthritis on most days? By most days, we mean more than half the days of the month.”, with separate questions for: acetaminophen, over-the-counter NSAIDs, prescription NSAIDs, prescription COX-2 inhibitors, doxycycline, prescription “strong pain” medications such as opioids, and knee injections of corticosteroid or hyaluronic acid. Self-report use of any of the abovementioned medications was considered using conventional medication.

To analyze CAM and conventional medication use simultaneously, we categorized use of treatment approaches into four groups, i.e., using only CAM, using only conventional medications, concomitantly using both and using neither (reference group).

### Potential correlates

Besides race, we considered other sociodemographic and clinical characteristics which may potentially influence using treatment approaches. Trained interviewers administered surveys which collected self-reported information on sociodemographic characteristics, including age, gender, education, income, marital status and insurance coverage for prescription medicines. Participants were asked to recall the highest grade that they had completed, with options including high school graduate, some college degree or technical school and college graduate or above. Income was measured with personal family income for the last year, including all sources such as wages, salaries, social security and retirement benefits. Options for this question were: ≤ $50,000, $50,000–100,000 and > $100,000. Participants’ marital status was categorized as either being married/partnered or being single (i.e., widowed, divorced, separated or never married). Participants were considered having prescription insurance coverage if they gave positive answers to the question “Do you have any health insurance plan that pays for all or part of the cost of prescription medicines?”

OAI used Knee injury and Outcomes in Osteoarthritis Score (KOOS) [24] to measure the severity of OA-related knee symptoms. There are five domains for KOOS scale, i.e., Pain, Symptoms, Knee Related Quality of Life, Function in Daily Living and Function in Sport and Recreational Activities. For each domain, a summary score ranging from 0 to 100 was calculated. Score 0 indicates extreme symptoms and 100 indicates no symptoms.

Symptom-related multi-joint OA was measured to capture the effect of generalized OA on using treatment approaches. We considered symptom-related multi-joint OA present if participants had OA symptoms in any of the three joints: hand, hip and lower back. We also considered the history of having a knee injury that limited ability to walk for at least two days, and history of having knee surgery that included arthroscopy, ligament repair or meniscectomy.

The 12-item Short-Form Health Survey (SF-12) provided an assessment of general health status [25]. Answers to the 12 questions were combined, scored, and weighted to create physical health score and mental health score. The scores also range from 0 to 100, with greater value indicating better health status. Body mass index (BMI) was calculated from measured height and weight [weight (kg)/height (m^2^)]. Participants with a BMI greater than 30 kg/m^2^ were considered obese [26].

### Statistical analysis

Our first goal was to evaluate the extent to which treatment approaches differed by race. To do this, we first evaluated the distribution of the outcome variable (CAM and conventional use simultaneously) and then deconstructed the composite measure to look at the specific distributions of conventional medications and CAM approaches by race. Because we were concerned about confounding by sociodemographic and clinical characteristics, we developed a multivariable multinomial logistic regression model to estimate the association between race and treatment use [27]. We first visually inspected the distribution of sociodemographic and clinical characteristics for AA and CA. We considered variables with absolute differences of greater than 5% as potential confounders [28]. Before modeling we also evaluated the potential for multicollinearity amongst the possible correlate variables under study by checking correlations between the covariates. Because KOOS Pain subscale was highly correlated with subscales of Symptoms, Function in Daily Living and Function in Sport and Recreational Activities (correlation coefficients were over 0.85), only Pain and Quality of Life subscales were included in the modeling.

An iterative approach (but not computer-driven) was used to build the model. We first entered each potential confounder separately into the model with the term for race (coded as 1 for AA, 0 for CA). From these models, we selected the factor that altered the effect for race the most and retained this variable for the next iteration. We then considered additional candidate variables. We proceeded until no additional variables induced material change (at least 10%) to our estimate of effect. To assure ourselves that additional groups of variables combined did not introduce confounding, we also developed an adjusted model which included a cluster of sociodemographic variables and then added a cluster of clinical characteristics. The models produced three odds ratios (ORs) and corresponding 95% confidence intervals (CIs) to compare AA to CA: 1) odds of using CAM only relative to use of neither; 2) odds of using conventional medications only relative to use of neither; and 3) odds of using both CAM and conventional medications relative to use of neither.

To describe the correlates of treatment use patterns among AA and CA, we developed separate multinomial logistic regression models by race. Correlates considered included sociodemographic and clinical characteristics. Although initially evaluated, medication insurance coverage, marital status, SF-12 mental health score, KOOS Function in Daily Living and KOOS Function in Sport and Recreational Activities were not associated with treatment use in CA or AA. Thus, we did not include these factors in the final models. To provide more clinically meaningful results for the KOOS subscales and SF-12 physical health score, we provided ORs of one standard deviation change in each of these variables. The number of AA in our analytic sample was substantially smaller than that of CA (508 versus 2,075) and as such, some correlates may not have reached statistical significance owing to limited sample size. Recognizing the limitations of p-values in this situation, we considered statistically significant correlates among CA whose ORs were identical or qualitatively similar to the ORs among AA as noteworthy.

## Results

Table 1 shows the sociodemographic and clinical characteristics between AA and CA with radiographic-confirmed OA of the knee. The gender distribution varied with more AA being women than CA. AA were younger, less likely to be married or in partnered relationships, had attained less education, and had lower income than CA. There were a higher proportion of persons being obese among AA relative to CA. Compared to AA, CA reported better outcomes on five domains of KOOS scale.

**Table 1:** Characteristics of participants with radiographic-confirmed knee osteoarthritis by race (N = 2583)

**Characteristic**	**African Americans**	**Caucasian Americans**
	**(n = 508)**	**(n = 2075)**
	*percentage*	
Age (years): ≥ 65	30.5	46.4
Women	70.9	55.0
**Education**		
≤ High school	31.0	15.3
≥ Some college	69.0	84.7
**Income ($)**		
≤ 50,000	66.5	36.9
50,000–100,000	25.9	38.2
>100,000	7.6	24.8
Married/partnered	37.2	72.7
Insurance covers prescriptions	84.6	87.5
Kellgren-Lawrence Grade		
2	33.5	31.5
3	49.4	47.2
4	17.1	21.3
Symptom-related multi-joint osteoarthritis	68.5	68.9
History of knee injury or surgery	46.9	56.3
Obesity	66.1	38.5
	*mean (standard deviation)*	
KOOS Pain	61.6 (23.0)	78.3 (18.1)
KOOS Symptoms	63.7 (21.6)	78.6 (17.6)
KOOS Quality of Life	51.2 (23.4)	65.5 (22.1)
KOOS Function in Daily Life	67.9 (22.3)	84.8 (16.2)
KOOS Function in Sports and Recreational Activities	52.8 (24.3)	69.9 (21.5)
SF-12 Mental Health Score	52.1 (10.1)	54.3 (7.6)
SF-12 Physical Health Score	42.3 (10.8)	48.9 (8.7)

Shown in Table 2 are the specific CAM therapies used by AA and CA. Biologically based supplement use was more common among CA (35.4%) relative to AA (17.9%). Glucosamine and chondroitin use were the most commonly reported CAM supplement use among both CA and AA, but CA were three times as likely as AA to use them. The opposite pattern was observed for use of biologically based topical agents with AA (23.2%) reporting use more than CA (10.7%). Six percent of CA reported using chiropractic or massage, but it was rarely used by AA (1.4%). AA were more likely than CA to report relaxation therapies or spiritual activities (12.4% versus 4.7%). Table 3 shows the use of conventional medications as current treatments of OA by race. Over-the-counter NSAIDs were the most commonly used drugs to treat OA in both groups, and its use was more prevalent among AA (28.0%) than among CA (19.5%). AA were also more likely than CA to use acetaminophen (17.9% versus 9.5%).

**Table 2:** CAM use among participants with radiographic-confirmed knee osteoarthritis by race (N = 2583)

Category^*^	**African Americans**	**Caucasian Americans**
	**(n = 508)**	**(n = 2075)**
	*percentage*	
**Alternative medical systems**	**0.2**	**1.4**
Acupuncture/ Acupressure	0.2	1.0
Ayurveda/biofeedback/energy healing/ hypnosis/naturopathy/Homeopathy	0.0	0.7
**Mind-body interventions**	**13.8**	**9.7**
Yoga/Tai Chi/Chi Gong/pilates	2.8	6.5
Relaxation therapy, meditation, deep breathing or visualization, spiritual activities	12.4	4.7
**Manipulation and body-based methods**	**1.4**	**6.1**
**Energy therapies**	**3.7**	**3.5**
**Biologically based therapies: topical agents**	**23.2**	**10.7**
**Biologically based therapies: supplements**	**17.9**	**35.4**
Glucosamine (nearly every day)	11.6	31.7
Chondroitin (nearly every day)	10.4	29.0
Vitamins/minerals	5.5	6.4
Methylsulfonylmethane (MSM)	3.9	6.3
Herbs	3.0	1.2
S-adenosylmethionine (SAME)	0.4	0.5
**Biologically based therapies: diet**	**1.2**	**1.0**

*As defined by the National Center for Complementary and Alternative Medicine.

**Table 3:** Conventional medication use among participants with radiographic-confirmed knee osteoarthritis by race (N = 2583)

**Category**	**African Americans**	**Caucasian Americans**
	**(n = 508)**	**(n = 2075)**
	*percentage*	
Acetaminophen	17.9	9.5
Over-the-counter NSAIDs	28.0	19.5
Prescription NSAIDs	10.2	7.0
COX-2 inhibitors	5.7	9.3
Opioids	3.9	2.6
Knee injection	4.7	3.5
Corticosteroid	4.1	2.4
Hyaluronic Acid	0.6	1.3
Doxycycline	0.6	0.3

Table 4 shows the association between race and treatment approaches used for OA. Overall, 16.5% of AA and 24.2% of CA exclusively used CAM to treat OA, 25.0% of AA and 23.8% of CA used CAM in conjunction with conventional medications, and 24.8% of AA and 14.6% of CA exclusively used conventional medications. However, after adjustment for sociodemographic and clinical characteristics, AA were less likely to use CAM either alone or in combination with conventional medications. While in the unadjusted analysis AA were more likely than CA to exclusively use conventional medications, this was explained by confounding by disease severity.

**Table 4:** Association between race and using treatment approaches among participants with radiographic-confirmed knee osteoarthritis (N = 2583)

**Treatment Use**	**African Americans**	**Caucasian Americans**	**Crude**	**Sociodemographic Characteristics Adjusted**^**†**^	**Sociodemographic and Clinical Characteristics Adjusted**^‡^
	**(n = 508)**	**(n = 2075)**			
	*percentage*		*Odds ratios of African Americans relative to Caucasian Americans (95% confidence intervals)*		
CAM Only	16.5	24.2	0.76	0.77	0.68
			(0.57–1.01)	(0.56–1.06)	(0.48–0.96)
Conventional Medication Only	24.8	14.6	1.89	1.57	0.96
			(1.45–2.46)	(1.16–2.15)	(0.68–1.35)
Both	25.0	23.8	1.17	1.11	0.59
			(0.91–1.51)	(0.83–1.49)	(0.42–0.83)
Neither	33.7	37.5	Reference group of outcome variable		

^**†**^ Adjusted sociodemographics include age, gender, education, income, marital status and medication insurance coverage.

^‡^ Besides sociodemographic variables, full model also adjusted KOOS Pain and Quality of Life subscales, Kellgren-Lawrence Grade, history of knee injury or surgery, symptom-related multi-joint osteoarthritis, SF-12 mental health scale, SF-12 physical health scale and obesity status.

The correlates of treatment use were identified for AA (Table 5) and CA (Table 6). Among AA, those aged 65 years or older tended to report more use of treatments relative to younger participants. Women were more likely to use CAM alone or in combination with conventional medications than men. An association was also found between higher incomes and using CAM. Among CA, women were more likely than men to use all treatment approaches. Income greater than $100,000 was associated with concomitantly using both types of treatments. Radiographic evidence of severe knee joint damage was correlated with using all treatment approaches. Symptoms measures, including knee pain, knee-related quality of life, symptoms-related multi-joint OA and general physical health, were also correlated with using CAM or conventional medications.

**Table 5:** **Correlates**^**†**^** of treatment approaches among African Americans with radiographic-confirmed knee osteoarthritis (N = 508)**

	**CAM Only (N = 84)**	**Conventional Medication Only (N = 126)**	**Both (N = 127)**
	*Odds ratios (95% confidence intervals)*		
Age ≥ 65 years	1.74 (0.90–3.37)	1.44 (0.80–2.61)	2.03 (1.09–3.77)
Women	1.79 (0.91–3.53)	1.02 (0.57–1.80)	1.82 (0.96–3.43)
**Education**			
≥ Some college	1.02 (0.50–2.07)	0.96 (0.53–1.76)	1.55 (0.81–2.96)
High school or less	1.0	1.0	1.0
**Income ($)**			
>100,000	2.23 (0.72–6.92)	0.47 (0.12–1.86)	2.82 (1.01–7.84)
50,000–100,000	2.28 (1.15–4.50)	0.95 (0.50–1.82)	0.83 (0.41–1.68)
≤ 50,000	1.0	1.0	1.0
KOOS Pain^‡^	0.78 (0.50–1.23)	0.65 (0.44–0.97)	0.54 (0.35–0.81)
KOOS Quality of Life^‡^	0.81 (0.52–1.25)	0.71 (0.49–1.05)	0.66 (0.44–1.00)
Kellgren-Lawrence grade:	0.94 (0.41–2.17)	0.91 (0.44–1.90)	1.08 (0.52–2.26)
4 versus 2 or 3			
Symptom-related multi-joint osteoarthritis	1.06 (0.56–2.00)	1.04 (0.59–1.85)	1.56 (0.83–2.93)
History of knee injury/surgery	0.93 (0.51–1.68)	0.64 (0.37–1.09)	0.77 (0.44–1.35)
SF-12 Physical Health Score^‡^	0.72 (0.48–1.09)	0.77 (0.53–1.10)	0.72 (0.49–1.04)
Obesity	1.05 (0.56–1.96)	1.18 (0.67–2.06)	0.71 (0.39–1.30)

^†^ Reference group includes participants who did not report use of CAM or conventional medications for osteoarthritis treatment.

^‡^ Odds ratios are per one standard deviation (SD) change in SF-12 Physical Health Score (SD = 10.8) and KOOS subscales of Pain (SD = 23.0) and Quality of life (SD = 23.4).

**Table 6:** **Correlates**^**†**^** of treatment approaches among Caucasian Americans with radiographic-confirmed knee osteoarthritis (N = 2075)**

	**CAM Only**	**Conventional Medication Only**	**Both**
	**(N = 502)**	**(N = 303)**	**(N = 493)**
	*Odds ratios (95% confidence intervals)*		
Age ≥ 65 years	1.07 (0.82–1.38)	0.98 (0.72–1.34)	1.10 (0.83–1.45)
Women	1.67 (1.30–2.15)	1.54 (1.14–2.08)	2.36 (1.79–3.11)
**Education**			
≥ Some college	1.81 (1.23–2.67)	0.89 (0.60–1.32)	1.25 (0.85–1.83)
≤High school or less	1.0	1.0	1.0
**Income ($)**			
>100,000	0.86 (0.61–1.22)	1.15 (0.76–1.75)	1.47 (1.02–2.12)
50,000–100,000	0.83 (0.62–1.11)	0.97 (0.68–1.37)	0.97 (0.70–1.33)
≤ 50,000	1.0	1.0	1.0
KOOS Pain^‡^	1.09 (0.90–1.33)	0.78 (0.63–0.97)	0.75 (0.62–0.91)
KOOS Quality of Life^‡^	0.69 (0.57–0.83)	0.87 (0.69–1.09)	0.59 (0.48–0.73)
Kellgren-Lawrence grade:	1.62 (1.18–2.24)	1.45 (1.00–2.11)	1.90 (1.37–2.65)
4 versus 2 or 3			
Symptom-related multi-joint osteoarthritis	1.43 (1.11–1.85)	1.67 (1.21–2.30)	2.43 (1.79–3.29)
History of knee injury/surgery	1.40 (1.09–1.81)	1.27 (0.93–1.72)	1.06 (0.80–1.39)
SF-12 Physical Health Score^‡^	0.98 (0.84–1.15)	0.68 (0.57–0.81)	0.75 (0.64–0.88)
Obesity	1.14 (0.89–1.47)	0.78 (0.58–1.05)	1.10 (0.84–1.44)

^†^ Reference group includes participants who did not report use of CAM or conventional medications for osteoarthritis treatment.

^‡^ Odds ratios are per one standard deviation change in SF-12 Physical Health Score (SD = 8.7) and KOOS subscales of Pain (SD = 18.1) and Quality of life (SD = 22.1).

## Discussion

In a large population of participants with radiographic-confirmed OA of the knee, overall CAM use is prevalent, but less so among AA. After adjustment for sociodemographic and clinical characteristics, CAM use was less common among AA than CA, while no differences in use of conventional medications remained. In addition to differences in the overall prevalence of use, we observed differences in the specific types of CAM used by race. The cross-sectional data used in this study do not permit any evaluation of the effectiveness of CAM approaches on symptom management in knee OA, nor do we suggest these data are documenting health disparities. Rather, the unique data collected as part of the OAI (symptoms, severity, radiographic confirmation, detailed CAM assessments) are useful to further our understanding of racial differences in CAM use.

Our finding that AA are less likely to use CAM therapies than CA is consistent with some previous studies [13,29,30]. Our work extends similar reports based on the general US population [29,30], as well as persons with self-reported arthritis [13], to a population with radiographically confirmed knee OA. Our work is not consistent with other reports [14,17], however. Comparing results of CAM use across studies is challenging because researchers often use different definitions of CAM. The detailed questionnaire of the OAI related to CAM permitted us to place our study into context with the extant literature. We confirmed that indeed the discrepancies between our findings and results from several other studies may primarily be due to different definitions of CAM use. Katz and Lee reported that CAM was more common among AA than CA, which was driven by biologically based diets to treat OA (75.2% of AA used biologically based diet relative to 60.9% of CA) [14]. In our study, however, we found only 1% of AA and CA used this method. Another study reporting greater CAM use among AA relative to CA did not include massage and chiropractic service as CAM therapy [17]. Consistent with other studies [13], we found that CA used more biologically based supplements, especially glucosamine and chondroitin, and chiropractic services relative to AA. Also consistent with the literature [14,16,17], we found that AA used more spiritual and religious activities and topical agents relative to CA.

We found that, without adjusting sociodemographic and clinical characteristics, AA were nearly twice as likely as CA to use conventional medications. Greater scrutiny of the actual medications revealed that the largest differences were observed with over-the-counter medications such as acetaminophen and NSAIDs, whereas CA reported greater use of prescription COX-2 inhibitors relative to AA (9.3% versus 5.7%). This is consistent with previous work [18,19], as well as in equal access health systems requiring minimal co-payments for medications [31]. In our study and in others [31,32], AA experienced more severe pain and other symptoms than CA despite having less severe radiographic evidence of disease. Nevertheless, after controlling for clinical factors, we found similar use of conventional medications by race. Yet, despite these adjustments, the finding that AA were less likely than CA to use CAM either exclusively or with conventional medications persisted.

While the reasons for racial differences in CAM use are likely multifactorial, one possible reason for the differences we observed may be differential access to care by race. Compared to AA, CA have better access to health care services [33]. In equal access systems, no racial differences in overall frequency of OA related physician visits or visits to rheumatologists were observed among veterans with OA [31]. But in our study, CA were more likely to have reported X-ray examination of their knees prior to study entry relative to AA which may in turn have led to more treatment using CAM. Moreover, relative to AA, CA did appear to report more CAM approaches that required access to the medical system (practitioners), e.g., chiropractic service. Further, in our study, AA had less favorable socioeconomic positioning (as measured by marital status, education and income) relative to CA. CAM therapies are not covered by insurance. Indeed, among AA, those with income over $50,000 were more likely to use CAM therapies. Some of CAM approaches, such as glucosamine [34] and acupuncture [35], have been shown beneficial in relieving symptoms among OA patients. However, as found in our study, both glucosamine and acupuncture were less frequently used among AA than among CA. If further studies confirm the effectiveness in delaying disease progression and ameliorating symptoms, CAM therapies should be promoted and made accessible to minority populations.

We found an association between age and CAM use among AA, but not CA. Older AA were more likely than those younger than 65 years of age to report use of CAM, conventional medications or both, but this age effect was not found among CA. Our findings align with studies that report older AA hold more positive views about CAM and use CAM more frequently than younger AA [15,36]. The differential effects of age on using conventional medication may be explained by different perceptions between AA and CA on side effects of conventional medications [31]. AA are less likely than CA to recognize any risk associated with over-the-counter and prescription NSAIDs [37]. Indeed, AA are less likely than CA to report that their doctors have discussed NSAID-related gastrointestinal problems [37]. Given that NSAIDs constitute the majority of conventional medications used for treating OA, this could explain why there was no increase in conventional medication use among older CA compared to younger CA.

Our study has several limitations. First, we could only evaluate CAM practices in AA and CA. While others report that Hispanics and Asian Americans are more likely to use CAM therapies [14,38], we were unable to evaluate this in our data. Second, it would have been preferable to identify correlates for each type of CAM treatment, but we were unable to because of inadequate sample size. Such analyses would have been more useful for understanding potential areas for intervention [39]. Lastly, only self-reported information on CAM and conventional medication therapies was available. Use of treatments was based on a 30-day or 6-month recall. As such, it is possible that participants did not accurately report the use of treatments. Further, persons with OA of the knee are prone to recall bias of treatments [40]. Nevertheless, if present, the misclassification of participants to treatment approaches is likely to have been non-differential which would have diluted any observed associations.

## Conclusions

We observed racial differences in use of CAM therapies either alone or in conjunction with conventional medications, but similar use of exclusive conventional medications by race. Understanding the extent to which the observed differences represent overuse of CAM among CA with OA of the knee, or underuse of CAM among AA with OA of the knee is a critical next step. With respect to correlates of use of treatment approaches, older AA are more enthusiastic about trying both CAM and conventional medications than younger AA. Socioeconomic disadvantages of AA may explain their less use of CAM compared to CA. More studies are needed to explore the benefits and risks of both conventional and CAM therapies among the underserved populations.

## Abbreviations

AA: African Americans; BMI: body mass index; CA: Caucasian Americans; CAM: complementary and alternative medicine; CI: confidence interval; KOOS: Knee injury and Outcomes in Osteoarthritis Score; NSAIDs: non-steroidal anti-inflammatory drugs; OA: osteoarthritis; OAI: Osteoarthritis Initiative; OARSI: Osteoarthritis Research Society International; OR: odds ratio; SF-12: 12-item Short-Form Health Survey.

## Competing interests

The Osteoarthritis Initiative (OAI) is a public-private partnership comprised of five contracts (N01-AR-2-2258; N01-AR-2-2259; N01-AR-2-2260; N01-AR-2-2261; N01-AR-2-2262) funded by the National Institutes of Health, a branch of the Department of Health and Human Services, and conducted by the OAI Study Investigators. Private funding partners include Pfizer, Inc.; Novartis Pharmaceuticals Corporation; Merck Research Laboratories; and GlaxoSmithKline. Private sector funding for the OAI is managed by the Foundation for the National Institutes of Health.

Dr. Eaton has received grants from and has served as a consultant to Pfizer. Dr. Lapane has served as a consultant to Pfizer and Ortho McNeil Johnson.

## Authors’ contributions

All the authors made material contribution to the completion of this manuscript. KLL contributed to the conception and design of this study, interpretation of the data, and writing of the manuscript. SY analyzed and interpreted the data and wrote the first draft of the manuscript. RJ, TEM and CBE were involved in interpretation of the data and critical revision of the article for important intellectual content. All authors gave final approval of the article.

## Pre-publication history

The pre-publication history for this paper can be accessed here:

http://www.biomedcentral.com/1472-6882/12/86/prepub
